# The Spatio-Temporal Distribution of Particulate Matter during Natural Dust Episodes at an Urban Scale

**DOI:** 10.1371/journal.pone.0160800

**Published:** 2016-08-11

**Authors:** Helena Krasnov, Itai Kloog, Michael Friger, Itzhak Katra

**Affiliations:** 1 Department of Geography and Environmental Development, Ben-Gurion University of the Negev, Beer-Sheva, Israel; 2 Department of Public Health, Faculty of Health Sciences, Ben-Gurion University of the Negev, Beer-Sheva, Israel; Coastal Carolina University, UNITED STATES

## Abstract

Dust storms are a common phenomenon in arid and semi-arid areas, and their impacts on both physical and human environments are of great interest. Number of studies have associated atmospheric PM pollution in urban environments with origin in natural soil/dust, but less evaluated the dust spatial patterns over a city. We aimed to analyze the spatial-temporal behavior of PM concentrations over the city of Beer Sheva, in southern Israel, where dust storms are quite frequent. PM data were recorded during the peak of each dust episode simultaneously in 23 predetermined fixed points around the city. Data were analyzed for both dust days and non-dust days (background). The database was constructed using Geographic Information System and includes distributions of PM that were derived using inverse distance weighted (IDW) interpolation. The results show that the daily averages of atmospheric PM_10_ concentrations during the background period are within a narrow range of 31 to 48 μg m^-3^ with low variations. During dust days however, the temporal variations are significant and can range from an hourly PM_10_ concentration of 100 μg m^-3^ to more than 1280 μg m^-3^ during strong storms. IDW analysis demonstrates that during the peak time of the storm the spatial variations in PM between locations in the city can reach 400 μg m^-3^. An analysis of site and storm contribution to total PM concentration revealed that higher concentrations are found in parts of the city that are proximal to dust sources. The results improve the understanding of the dynamics of natural PM and the dependence on wind direction. This may have implications for environmental and health outcomes.

## Introduction

Dust storms, a common phenomenon in arid and semi-arid areas, have significant impacts on both the physical and human environments due to the chemical [1–2] and biological [3–4] properties of the dust particles carried by the storm winds. Studies have shown that atmospheric dust particles can have marked effects on human health [3, 5–9], especially during dust storm episodes when the amounts of airborne particulate matter (PM) are much higher.

As one of the principal air pollutants in the urban environment, PM has been extensively studied [10–12]. Several such studies have shown that dust storm events significantly increase PM levels to values above the ambient air quality standards of most countries [13–18]. In Arizona, approximately 79% of PM_10_ is desert dust-derived [19]. Rodriguez et al., [18] showed that PM_10_ values in Spain increased during episodes of high-dust Saharan air mass transport, with approximately 30% of the daily exceedance the European Directive limit for PM_10_ (50 μg m^-3^). In Taiwan, Chen et al., [20] showed higher PM_10_ levels (by about 68 μg m^-3^) for dust days compared to the average PM_10_ on non-dust days. A study by Kuo and Shen [21] showed that during Asian dust storms, the concentrations of both PM_2.5_ and of PM_10_ increased significantly. Krasnov et al. [22] showed that the daily net contribution of desert-derived dust to PM_10_ values in northern Israel was 122 μg m^-3^. Kassomenos et al. [23] revealed that in Greece the non-combustion-related fraction ranged between 25.1 and 72.7%, depending on the site and the season.

In recent years, an appreciation for the potentially marked variability of within-city air pollution has driven interest in assessing pollution at the intra-urban scale [11, 24–29]. Most cities, however, lack sufficient number of PM monitoring sites to enable them to identify intra-urban pollutant variability, making it difficult to gather findings on total population exposure [30]. In addition, most studies of PM spatial distribution at the city scale focus on anthropogenic sources of PM, while information on the spatial distribution of dust storm-derived PM in urban environments is limited. A study by Kassomenos et al. [31] estimated that the contribution of non-combustion sources varies substantially among cities, sites and seasons and ranges between 38–67% and 40–62% in London, 26–50% and 20–62% in Athens, and 31–58% and 33–68% in Madrid, for both PM_10_ and PM_2.5_

Past research has employed a variety of approaches, such as regression models [11–12, 32–34], aerosol optical depth measurements [35–36], geostatistics [37–40] and, increasingly in recent years, mobile monitoring [11–12, 41–42], but the resolution of these large-scale methods cannot detect local variation. Identifying and quantifying differences in pollution levels (natural and anthropogenic) on a smaller scale can lead to a better understanding of PM distribution and exposure.

High-resolution monitoring and mapping can contribute significantly to air quality management in terms of both pollution abatement and exposure and risk assessment. Air quality has improved greatly in North America and Europe in recent years, resulting in many health benefits [43–45]. In other parts of the world, specifically rapidly developing economies (especially those in arid environments exposed to dust storms) have not experienced such improvements in health. According to the World Health Organization (WHO), global population-weighted exposure to PM_2.5_ increased by 8% from 1990 to 2010, and 89% of the world’s population is exposed to annual average PM_2.5_ concentrations above the WHO air quality guideline of 10 μg m^−3^ [44] While the highest exposures occur in rapidly developing economies, the majority of outdoor air pollution research has been conducted in developed countries, which usually have large numbers of monitoring stations and whose levels of air pollution are typically relatively low.

The aim of this study, therefore, was to analyze the urban spatial-temporal behavior of dust storm derived PM in the city of Beer-Sheva in southern Israel. The results of this study may allow us to identify hot spots within Beer-Sheva where people may be at higher risk due to the seasonal intrusion of dust into that area. Quantitative data on particulate matter from arid areas and information on the spatial and temporal variation within arid cities is sparse. The choice of Beer-Sheva was motivated by the fact that this city is located in an arid area (which promotes the generation of soil-derived suspended particles) and because it has only one monitoring station. Real-time measurements were collected and interpolated using an inverse distance weighted (IDW) model to analyze the spatial differences between two types of days: non-dust (background) and dust days. We used these data to examine the spatial relationships at the city scale between dust storm-derived PM_2.5_ and PM_10_ concentrations.

## Materials and Methods

### Study area

Located in the Negev desert in southern Israel, the city of Beer-Sheva (31.2498° N, 34.7997° E; 117.5 km^2^) has hot, dry summers dominated by daily land and sea breeze circulation and wet, cool winters with considerable cyclonic activity. The Persian trough that dominates the summer synoptic meteorology generates ample sunshine, stable PM conditions and vigorous northwesterly winds on a daily basis from mid-May to October. During winter, the generally calm southeasterly flow is interjected with northwesterly subtropical storms which pass through the area at a frequency of about one per week. During the fall (and sometimes in the winter), the synoptic system of the Red Sea trough can cause periods characterized by dense desert dust and elevated PM levels.

The semi-arid northern Negev desert has an average annual rainfall of about 100 mm, most of which falls from December to March. The region’s average annual temperature is 18.3°C, with large differences between winter and summer and between day and night. Maximum average daily temperature is about 32°C from May through September, and the minimum may drop to freezing during January and February. Atmospheric humidity varies between 5% and 93%.

### PM data

Air quality data were collected from the single monitoring station set up by the Israel Ministry of Environmental Protection (www.sviva.gov.il) in Beer-Sheva. The station is situated on the roof of a building in the middle of the city (34.78132°N, 31.25674°E). This monitor is the sole monitoring station in the area monitoring ambient air quality. In addition, it also records other meteorological parameters: PM, wind speed and direction, air temperature, relative humidity, and concentrations of pollutants (SO_2_, CO, O_3_, NO, NO_2_). The PM data are recorded every 5 min by a dichotomous ambient particulate monitor (Thermo Scientific 1405-DF) for continuous direct measurements of PM by utilizing two tapered element oscillating microbalances (TEOM) calibrated using the volumetric air flow calibrator technique (National Institute of Standards and Technology, NIST).

### PM measurements

Mobile measurements were made using the TSI DustTrak DRX 8534 (TSI Inc., Shoreview, MN), which measures PM concentrations once per second. The DustTrak monitor was set to zero against a zero filter on each measurement day. The factory-specified resolution of the DustTrak monitor is ± 0.1% of the reading or ± 1 μg m^-3^, whichever is greater. All the necessary calibrations, including flow rate and zero tests, were carried out before every sampling tour, according to the manufacturer's instructions. The TSI DustTrak has been widely used in numerous studies of outdoor PM_2.5_ and PM_10_ due to its sensitivity to a range of different aerosols, fast response time, and a high temporal resolution of the measurements (despite having a well-known bias). Some studies corrected for this bias and obtained more accurate mass concentrations by operating the DustTrak simultaneously with a gravimetric sampler to obtain custom calibration factors [12, 45–48]. Other studies have simply relied on literature-based correction factors; for example, Branis and Vetvicka [48] divided their 15-min average DustTrak PM_10_ data by a factor of 2.5. A study by Jayaratne et al. [49] showed that the composition of the dust from dust storms is very similar to that of the Arizona Road Dust (ISO 12103–1, A1 test Dust) used to calibrate the DustTrak (TSI, 1997), thus instilling confidence that the DustTrak data provide a reasonably accurate measure of dust storm PM concentrations. The data from the DustTRak were not calibrated in this study but rather shown as the changes in magnitude of windblown dust with correlating conditions. The correlation between the DustTrak data and the stationary 1405-DF device show a good agreement of 85% (y = 0.99x-14.62), meaning that the actual concentration readings of the DustTrak monitor are sufficient to represent the impact of dust for the purpose of this study.

### Study design

As a first step, we analyzed the possible anthropogenic effect of the city that may influence PM concentrations. Time series data for the dust free months (July-August; Krasnov et al. [22]) were obtained from the Beer-Sheva monitoring station to evaluate PM temporal behavior. A curve presenting the daily (24 hours) and hourly changes of PM_2.5_ and PM_10_ during week days with anthropogenic activities (natural + anthropogenic) and weekends with less intense anthropogenic activities (natural) was produced. The results are compared to a monitoring station located in Tel Aviv (34.79488°N, 32.06645°E) in the center of Israel; about 100 km north to Beer-Sheva which is a large city in Israel, characterized by high anthropogenic PM sources.

**Fig 1:**
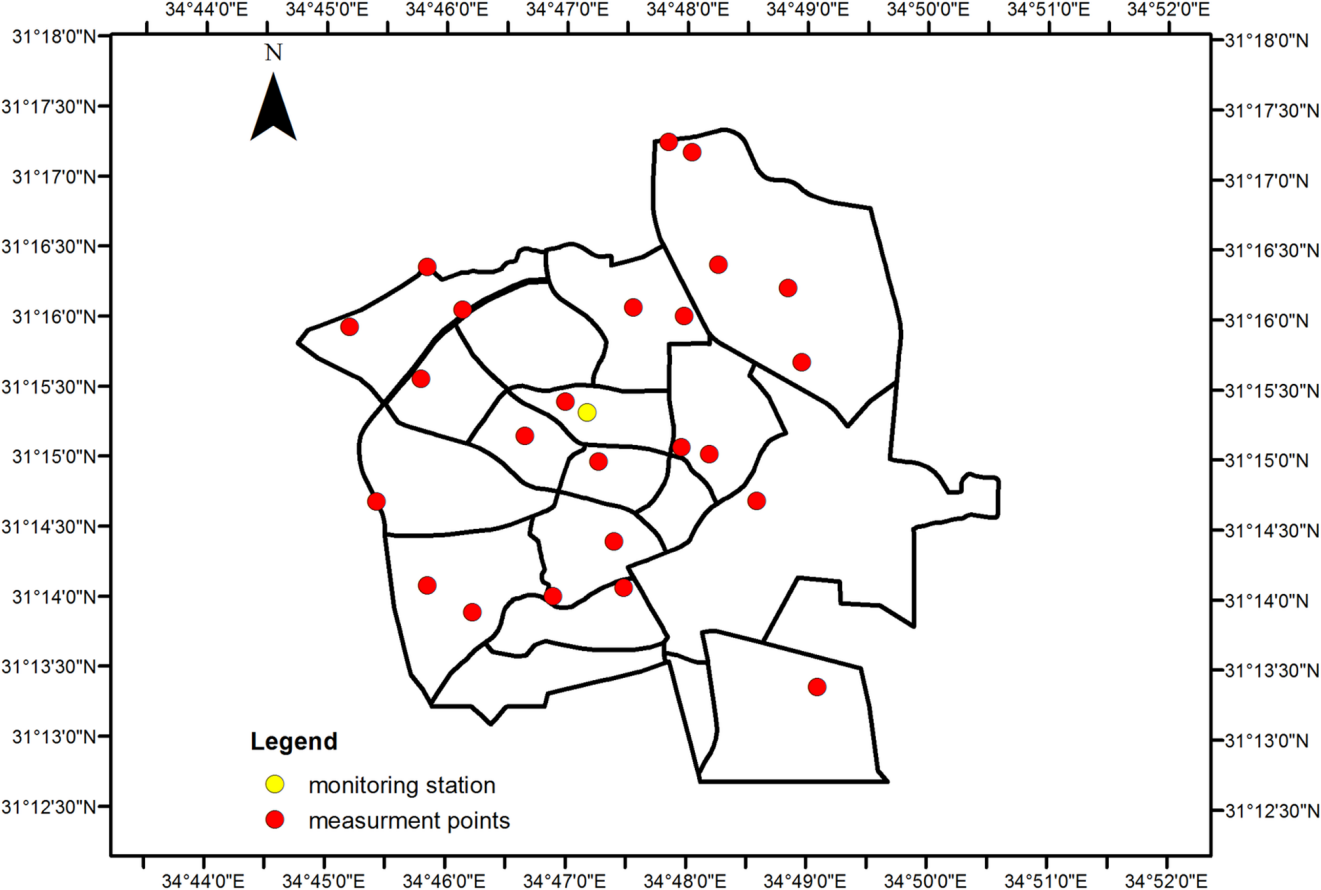
Map showing the locations of 23 measurement points (red dots) around the city of Beer-Sheva. Location of the Beer Sheva monitoring station is indicated by a yellow dot.

A study of spatial and temporal variability of air pollution would ideally be based on continuous data using a relatively dense network of monitors placed at multiple locations [45–46] and with additional information on dust sources and other factors gathered at each location. Such an approach would be prohibitively costly and logistically very difficult, because of issues such as power requirements and security of the equipment. Because substantial variability in pollution may occur over distances as small as 100 m, pollution data from a single monitoring site can only be considered representative of a small surrounding area. To obtain PM data on the whole-city scale, therefore, we used mobile monitors, a method that was previously used in studies by Dionisio et al., [11], Merbitz et al.,[33], and Levy et al [12]. These studies, however, evaluated anthropogenic sources of PM on a city scale, but did not look into natural dust derived sources. For spatial measurements, we separated the city into 16 neighborhoods, and the measurement points were distributed heterogeneously and placed in each neighborhood. For large neighborhoods, 2–3 measurement points were assigned (monitoring station of the Ministry of Environmental Protection in Beer-Sheva indicated by a yellow dot). Measurement points were placed more than 50 m away from main roads to avoid traffic bias and at each point, levels of PM_2.5_ and PM_10_ were obtained. A total of 23 ground level measurement points were set up throughout the city (Fig 1).

Measurement points were sampled during day time on two types of days: non-dust (background) and dust days, representing the different types of synoptic conditions prevailing in Israel. Sampling entailed making a two-hour drive on each sampling day from one measurement point to another along a predetermined path to each point in succession to ensure that all measurement locations were visited on the same day. Each site was sampled for 5–10 minutes (until PM levels were stable). To compensate for the temporal differences in the measurements, only data for days with low variability in PM concentration over the two-hour period (based on monitoring station data) were used. Mobile data collection for background days began randomly but not during the morning rush hour. Potential dust days were determined based on dust event prediction by the HCMR POSEIDON System (http://poseidon.hcmr.gr/) and defined as days on which PM_10_ values (from the Beer-Sheva monitoring station) were at least 100 μg m^-3^, which is above the threshold of dust storms in this area (71 μg m^-3^) [22]. Data for dust days were therefore collected on days when PM_10_ values were greater than 100 μg m^-3^. Wind flow during the dust episodes was calculated via 48-hour backward trajectories using the HYSPLIT 4 model of the Air Resources Laboratory of the NOAA [50] for three different levels: 500, 1000, 1500m.

The data for a total of 10 background sampling days (averaged because all non-dust days have similar characteristics) and seven dust event sampling days (i.e., the total number of storms during the sample year) were recorded. Information on meteorological conditions measured by the Beer-Sheva meteorological station during the sampling days is presented in Table 1. The data missing from the table show the drawback of trying to measure the area of an entire city with a single meteorological station.

**Table 1:** Beer-Sheva monitoring station data collected during measurement time. WS = wind speed; WD = wind direction; SS = synoptic system.

		WS(m s^-1^)	WD(°)	Temp (°C)	RH (%)	PM_10_ (μg m^-3^)	PM_2.5_ (μg m^-3^)	PM_2.5–10_ (μg m^-3^)	SS	Trajectory
**Background**	**Background**	2.62	171	16	58	42	24	19	(—)	(—)
**Dust days**	**January 26**	2.43	232	17	(—)	(—)	(—)	(—)	CL	West
	**February 22**	5.6	287	(—)	(—)	110	41	68	SL	West
	**March 2**	4.47	300	24	30	534	191	342	SL	West
	**March 6**	4.20	294	23	47	187	82	104	SL	West
	**March 17**	3.02	287	18	58	(—)	(—)	(—)	RST	West
	**April 7**	6.45	271	23	39	78	63	14	RST	West
	**May 5**	5.55	310	29	26	(—)	(—)	(—)	RST	West

Data collected for non-dust days (background) and dust days, including meteorological measurements (wind speed, wind direction, temperature, relative humidity) and PM levels. Synoptic system information provided by the Israel Meteorological Service.

RST = Red Sea Trough; SL = Sharav Low; CL = Cold Low; (—) = no data.

### Data processing

Geographic information system (GIS) tools were used to build a high-resolution geo-database to support the spatial analyses, which were performed using ArcGIS software version 10.1. The GIS database included land information for the city of Beer-Sheva. A 50-m resolution digital elevation model (DEM) comprising a digital terrain model (DTM) and a digital surface model (DSM), sub-areas and streets in the city were obtained from the Survey of Israel (MAPI) at a spatial resolution of 1 cm per pixel. Measurement point locations were added as separate layers in the system. Because of the small number of sample sites in our study, we used inverse distance weighted (IDW) interpolation, a detailed discussion of which can be found in Isaaks and Srivastava [51]. The IDW interpolation estimate is a linear combination of the observed values inversely weighted by the distances of the observation locations from the interpolation point. The IDW operator depends only on these distances and is independent of the observed values on which it operates.

To enable the quantitative comparison of the different maps, they were all produced at the same spatial scale and on the same grid. All the figures were produced using the same color palette. The colors were assigned to the concentration values according to a linear scale between the extreme concentration values on the map. Values were separated based on the IDW results using the default geometrical interval classification scheme. This enables a meaningful visual comparison of the features shown in the maps of the different types of days. Calculation of the root mean square error (RMSE) was used to evaluate spatial distribution map accuracy. It is a frequently used measure of the differences between values predicted by a model or an estimator and the values actually observed. A number of semivariograms were compared, and the best fit model was selected based on model fitness to the data.

At each site, PM_2.5_ and PM_10_ concentrations were characterized and compared using Pearson correlation coefficients, t-test, and univariate regression procedures. The relative contribution of PM_2.5_ particles to PM_10_ values was compared using PM_2.5_/PM_10_ concentration ratios. Results were considered significant when p < 0.05.

## Results

### General temporal PM behavior

**Fig 2:**
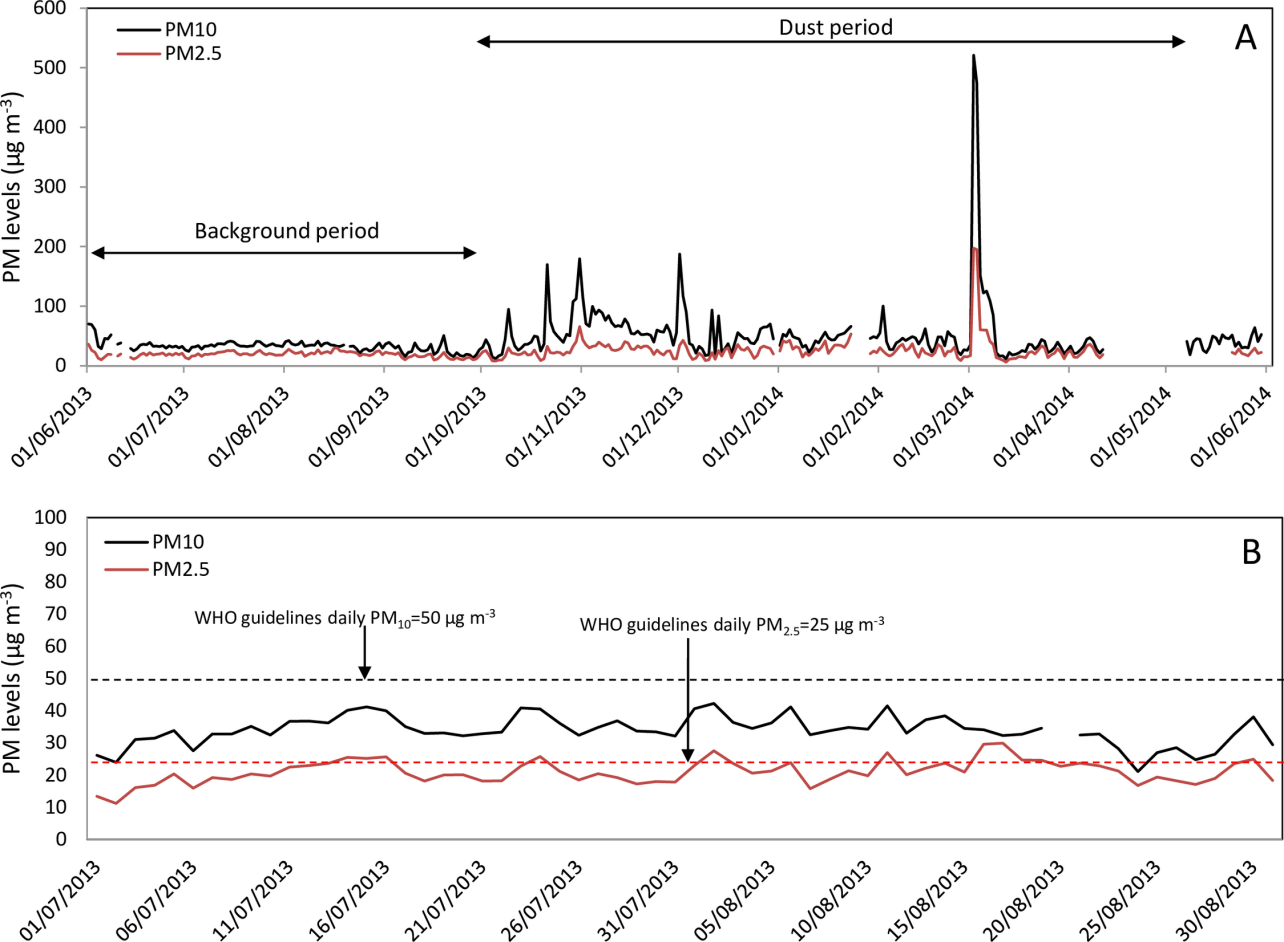
A. Daily average (24 hours) PM_10_ and PM_2.5_ concentrations (μg m^-3^) over one year (June 2013-June 2014) based on Beer-Sheva monitoring station data. B. Daily PM_10_ and PM_2.5_ concentrations (μg m^-3^) on background days in comparison with WHO limits.

Fig 2A presents daily (24 hours) PM average concentrations over one year (June 2013-June 2014) as measured by the Beer-Sheva monitoring station. The background period is characterized by a smooth curve with low concentrations, while the dust period is characterized by values with high amplitudes.

The daily PM_10_ concentrations on the background days remain within a narrow range (31–48 μg m^-3^; SD = 4.4) and do not exceed the WHO guidelines (Fig 2B). A similar trend was observed for PM_2.5_ although the concentrations are closer to the guideline value. These trends were evaluated for previous years as well. Overall, the results support the assumption that background measurements of PM are stable throughout the entire summer period regardless of the day of the month, and therefore, mobile data collection can begin randomly.

**Fig 3:**
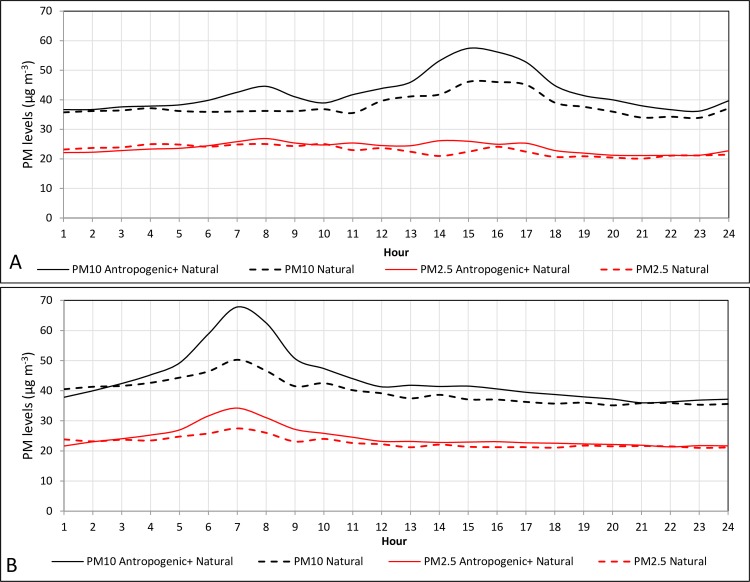
Hourly PM_10_ and PM_2.5_ concentrations (μg m^-3^) during the background period for Beer-Sheva (A) and Tel-Aviv (B) during weekday (continuous line) vs weekend (dashed line).

A closer analysis of the hourly PM concentrations during the background period show hourly trends in PM_10_ and PM_2.5_ for days with anthropogenic activities (natural + anthropogenic in Fig 3A, weekdays) and days without anthropogenic activities (natural, weekends and holidays). The anthropogenic activities induce a slight increase in PM_10_, from 38 μg m^-3^ to 45 μg m^-3^, during the morning rush hour period (07:00–09:00) and again during the afternoon (14:00–17:00), with a decrease after 17:00 (from 50 μg m^-3^ to 40 μg m^-3^). Note that the ‘natural’ PM_10_ curve does not increase during the morning hours, while the PM_2.5_ curve does not follow the afternoon trend. The difference between the two curves (days with vs. days without anthropogenic activity) reveals that the average anthropogenic contribution was less than 15% for both PM_10_ and PM_2.5_. For comparison, in Tel Aviv, a city with high anthropogenic activity (Fig 3B), both the PM_10_ and PM_2.5_ values increase when anthropogenic activities are at a peak between 06:00–09:00 (from 39 μg m^-3^ to 68 μg m^-3^ for PM_10_ and from 21 μg m^-3^ to 33 μg m^-3^ for PM_2.5_), but the afternoon increase in PM levels evident in Beer-Sheva was not observed in Tel Aviv.

**Fig 4:**
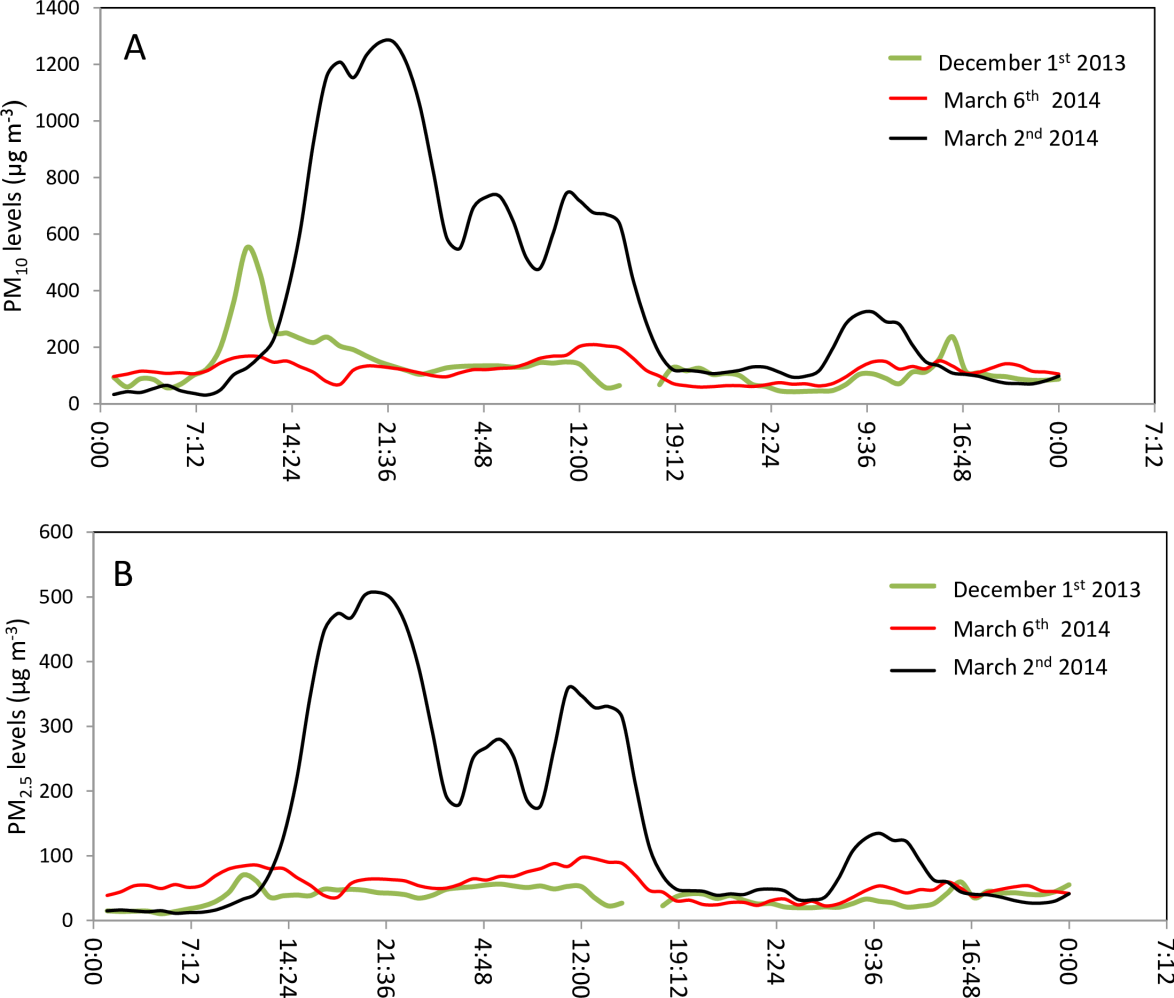
Hourly PM_10_ (A) and PM_2.5_ (B) concentrations (μg m^-3^) during three dust events.

During the dust period (November-May), storms vary in terms of their durations (hours) and PM concentrations. For example, in Fig 4 the storm of December 1, 2013 (green curve) has a narrow high peak of 550 μg m^-3^ for PM_10_ (Fig 4A), and 70 μg m^-3^ for PM_2.5_ (Fig 4B) and a duration of 38 hours. The storm of March 2, 2014 (black curve) has a high maximum hourly concentration (1280 μg m^-3^ for PM_10_, and 507 μg m^-3^ for PM_2.5_) with a duration of 55 hours, while the storm of March 6, 2014 (red curve) has a lower maximum hourly concentration (average 209 μg m^-3^ for PM_10_, and 70 μg m^-3^ for PM_2.5_) and a duration of 30 hours. Note that although the values of PM_2.5_ are lower than those of PM_10_, the two fractions exhibit similar trends.

### Averaged PM distributions

**Fig 5:**
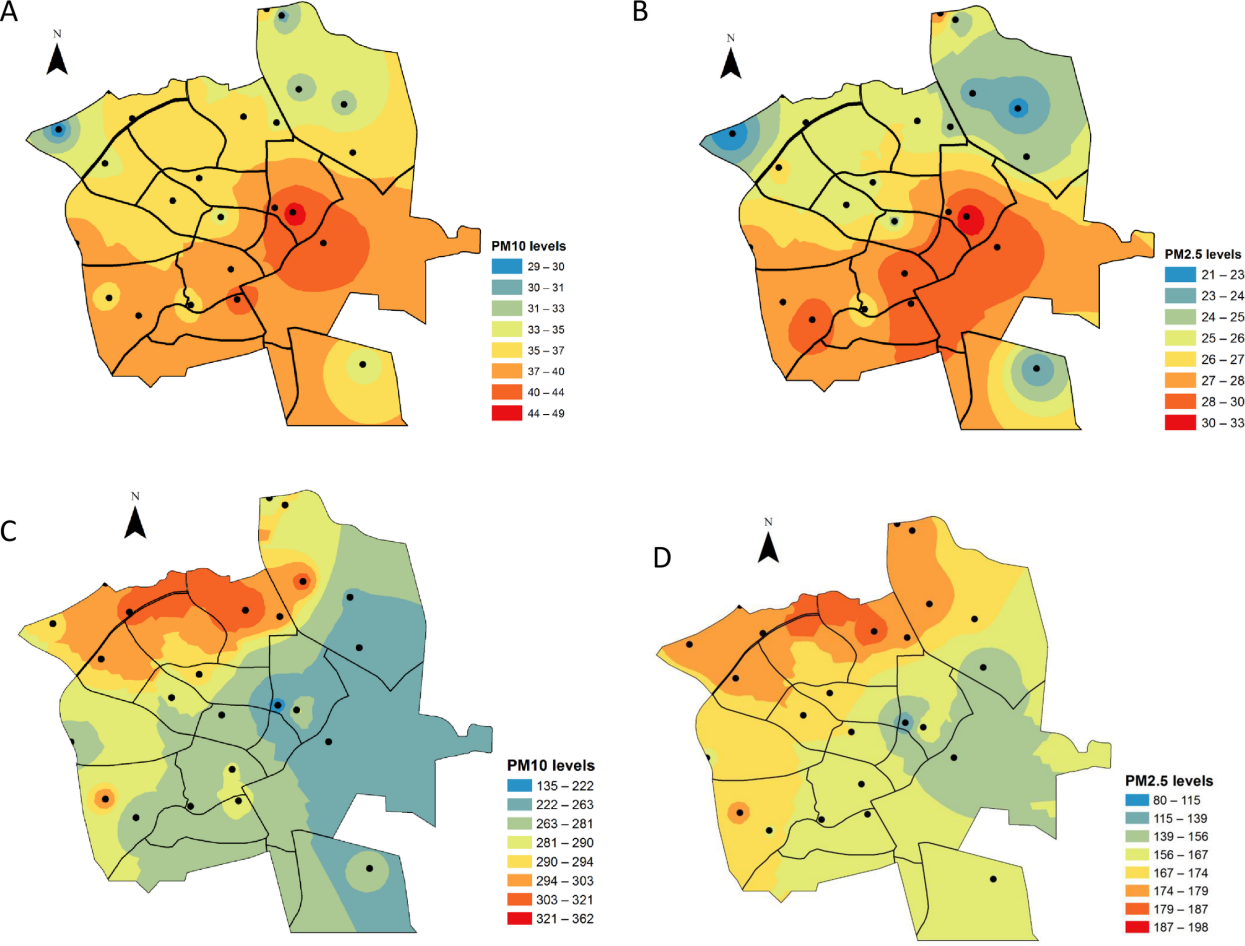
**A.** Average spatial distributions of PM for the background period: (A) PM_10_ concentrations (range: 29–49 μg m^-3^) and of (B) PM_2.5_ concentrations (range: 21–33 μg m^−3^). **B.** Average spatial distributions of PM for the dust period. (C) PM_10_ concentrations (range: 135–362 μg m^-3^), and (D) PM_2.5_ concentrations (range: 80–198 μg m^-3^).

Fig 5A and 5B show spatial distribution maps of the mean PM concentrations for the background vs. the dust storm periods. Also shown on each map are the locations of the stations whose data were used to produce the map.

Average spatial PM_10_ concentrations on background days were in the range of 29–49 μg m^-3^ (spatial SD = 4.8). These values, recorded at the mobile measurement points around the city, are in agreement with the PM_10_ values recorded at the Beer-Sheva monitoring station (Fig 2). Average PM_2.5_ concentrations were in the range of 21–33 μg m^-3^ (spatial SD = 3.1). The PM values measured in the southeastern and central part of the city were slightly higher than in the rest of the city (for both fractions), while those in the northwest were the lowest.

During the seven dust storm days, both PM fractions were elevated. The average PM_10_ concentrations increased from 135 to 362 μg m^-3^ (spatial SD = 45.8) while the average PM_2.5_ concentrations were in the range of 80 to198 μg m^-3^ (spatial SD = 24.3). The average distribution map of PM concentrations during the dust storm period revealed a trend opposite that reflected in the background map, i.e., higher PM concentrations were found in the northwestern part of the city.

A strong correlation was found between PM_2.5_ and PM_10_ values for the different measurement points for both types of day (Pearson coefficient = 0.94 for non-dust days; 0.91 for dust days), with PM_2.5_ comprising approximately 73% of PM_10_ (in the different neighborhoods) during background days and 64% during dust days. The RMSE for the background days ranged from 4.4 to1.8 for the two fractions, indicating that the averaged spatial distribution was a good representation of the background period. For dust days, however, the RMSE values (measure of the differences between values predicted by a model or an estimator and the values actually observed) for the averaged dust data were high–from 48 for PM_10_ to 25 for PM_2.5_ –which shows that the calculated average PM spatial distributions during dust storm events were not accurate representations of city dust distribution on stormy days. This finding was expected, since every dust storm behaves differently (Fig 4), thus dictating that each dust storm should be analyzed separately.

### Specific PM distributions during dust storms

**Fig 6:**
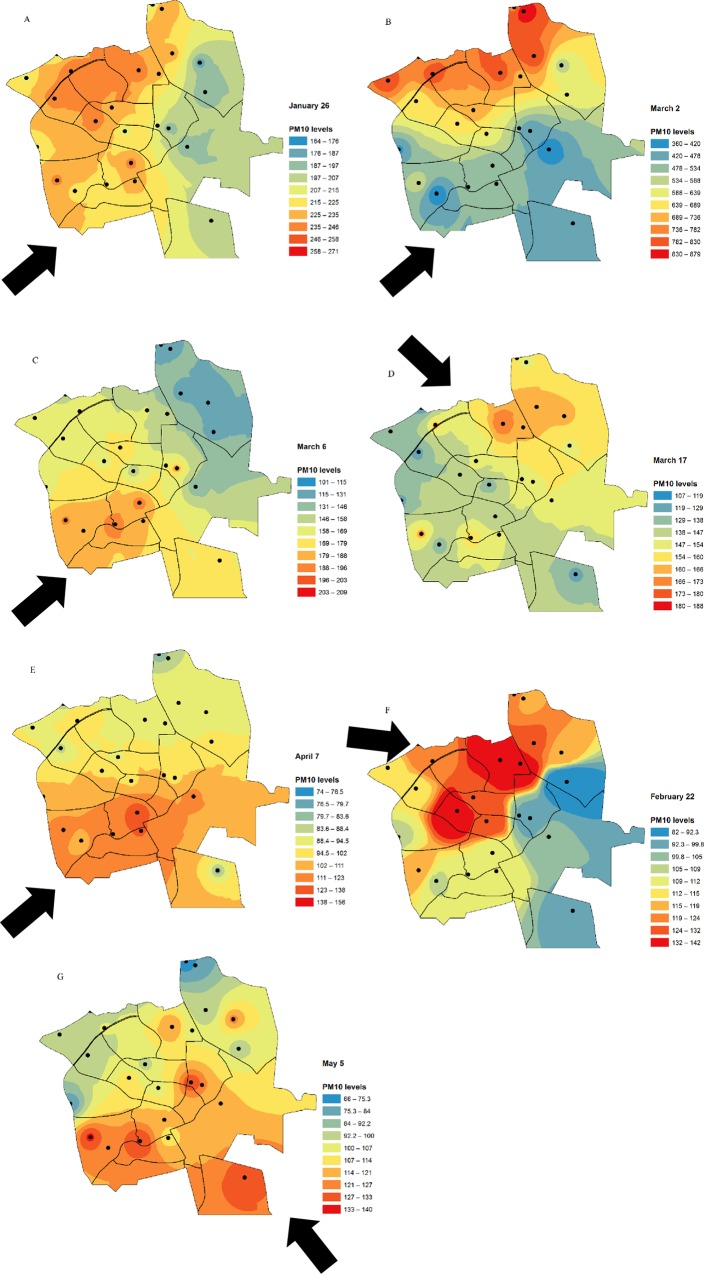
**A.** PM_10_ distribution maps for strong storms. Black arrows–Main wind trajectory during dust storm. Note PM concentration range. **B.** PM_10_ distribution maps for mild storms. Black arrows–Main trajectory of dust storm winds. Note PM concentration range.

Distribution models of PM_10_ concentrations for strong and mild dust storms (classification by Krasnov et al., [22]) are presented in Fig 6A and 6B, in which the predominant wind trajectory during each storm event is represented by a black arrow.

The strongest storm was that of 2 March, during which PM_10_ values exceeded 800 μg m^-3^ (16 times higher than the maximum background value), while PM measurements from the mildest storm were around 140 μg m^-3^ (2.5 times higher than the maximum background value, 5 May). The distribution models resulted from the interpolations show that the windward side of the city will have higher levels. This is true for six out of the seven storms.

A strong correlation was found between PM_2.5_ and PM_10_ values for the different measurement points during different storms (Pearson coefficient rages from 0.84 to 0.92). The PM_2.5_/PM_10_ ratio varied slightly between storms (SD~0.11) but not between measurement points (SD~0.04). The average PM_2.5_/PM_10_ ratio in the different neighborhoods was 0.6, and higher ratios were observed during the mild storms of 7 April (PM_2.5_/PM_10_ = 0.75 ± 0.12) and 22 February (PM_2.5_/PM10 = 0.88 ± 0.03).

### Neighborhood-specific PM concentrations

**Fig 7:**
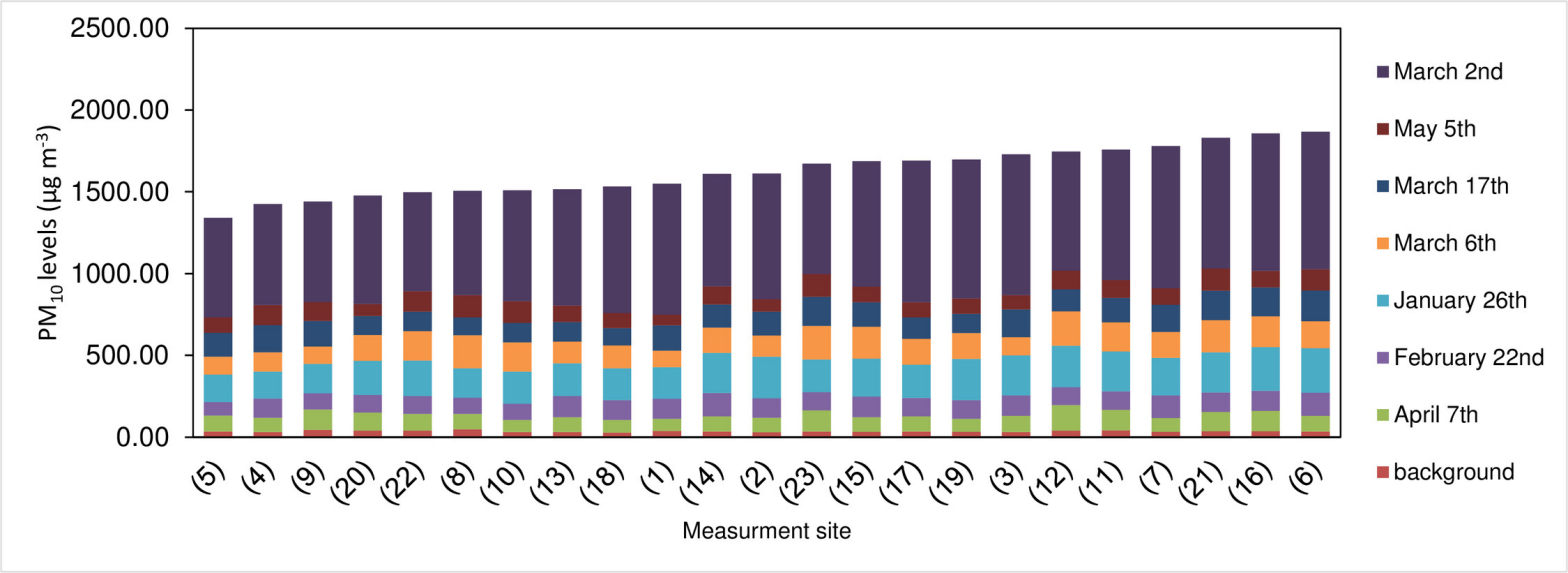
Total PM_10_ levels (lowest to highest arranged left to right) per measurement site over an entire dust season (December-April)

An analysis of total PM_10_ concentration per measurement site over an entire dust period is presented in Fig 7. As was previously shown, PM_10_ levels varied not only temporally by storm, but also spatially by neighborhood. The highest PM levels (above 1800 μg m^-3^) were measured at points 6, 16, and 21, all of which are located in the western part of the city inside residential areas. The lowest PM concentrations (below 1450 μg m^-3^) were measured at points 5, 4, and 9, which are located close to the city’s eastern boundary. The difference between the overall highest and lowest PM levels for the entire study period (measurement points 6 and 5, respectively) was 527 μg m^-3^.

## Discussion

### General temporal PM behavior

Hourly measurements of PM concentrations in Beer-Sheva showed that PM_10_ and PM_2.5_ levels increased during the morning hours on weekdays (i.e., days with high anthropogenic activity), but they remained relatively stable on weekends. This finding indicates that the morning peak (PM_10_ values of about 45 μg m^-3^) is caused by anthropogenic activities (e.g., traffic). Indeed, the Tel Aviv monitoring station exhibits a similar morning weekday peak but with much higher PM values (about 68 μg m^-3^ for PM_10_) indicative of the larger effect that anthropogenic pollution has in central Israel than in the northern Negev desert of southern Israel. Previous studies that have already shown this morning pattern and its connection to traffic [52–53] reported PM values similar to those observed in Tel Aviv. In addition to the morning peak, the hourly measurements also revealed an afternoon trend that was evident only in Beer-Sheva and only for the PM_10_ fractions. Because urban PM_2.5_ originates mainly from anthropogenic sources [52; 54–55] but remain stable, the noontime increase in PM_10_ is assumed to be related to natural sources. A similar trend was observed by Chow et al. [56] in California and explained by the late afternoon ventilation of the Southern California coast. In Beer-Sheva, southwesterly winds predominate during the afternoon and transport particles that are larger than 2.5 μm from the soils surrounding the city toward Beer-Sheva [57]. The afternoon peak is absent in Tel Aviv since the wind regime there is influenced by the Mediterranean Sea breeze, which blows during the day from the west and the northwest [58]. Likewise, a study by Querol et al. [52] attributed the similar absence of a late afternoon peak in Barcelona, Spain, to the thinning of the urban boundary layer. The results thus indicate that Beer-Sheva represents a unique setting whose total PM measurements have a relatively small anthropogenic contribution, making the city an ideal place to study natural dust.

The changes in PM_10_ and PM_2.5_ values that typically occur during dust storms are unpredictable, as each storm has a different effect on PM. Variations in PM_10_ concentrations due to dust storms were already shown via a ten-year analysis by Krasnov et al. [22].

### Averaged PM distributions

For days on which the background period was based, the PM_10_ and PM_2.5_ levels in the southeastern and central part of the city were higher than those in its northwestern section, which exhibited the lowest levels. This can be explained by the fundamentally different compositions of the two neighborhoods. The semi-commercial and semi-industrial southeastern and central part of the city contributes high levels of PM to the rest of the city. In contrast, the northern area of Beer-Sheva has higher topography and is relatively far from the city center, both of which promote better ventilation and reduced PM levels relative to other areas of the city. The strong correlation between PM_2.5_ and PM_10_ at the different measurement points indicates that regional meteorology has a dominant influence over local areas and a small spatial variability through the city during the background period. A study by Janssen et al. [59] showed that the variability in PM_10_ concentrations in non-arid environments is driven primarily by variability in PM_2.5_ concentrations, which comprise approximately 70% of PM_10_ around the world. Our results were similar for the background period (73%), and during dust events, that value decreased to 64%, which strengthens the understanding of the contribution of the PM_2.5–10_ fraction during dust intrusions.

A significant temporal variability was observed during the dust period compared to background period, which is expected and explained by the intrusion of dust storms to Beer-Sheva contributing net daily PM concentrations of up to 2,643 μg m^-3^ [22]. Dust storms increase PM_10_ concentrations in the city by an average factor of 8 compared to background days. The opposite trend of the averaged distribution map for the dust period is due to the fact that 6 out of 7 dust storms were a result of a west trajectory of the air mass transported to the region (Table 1).

### Specific PM distributions during dust storms

PM measurements revealed that each storm generated a different PM distribution map (Fig 6A and 6B), the formation of which is driven by the varied behavior of each storm. For most storms, the city-wide distribution of PM is affected by the trajectory of the wind, which also contributes particles emitted from soils proximal to the city. Although stronger storms increase PM levels in all the city’s neighborhoods, those on the windward side of the city will have levels that are far in excess of the WHO guidelines. In contrast, mild storms will cause slightly smaller increases in PM concentrations, such that the concentrations in some neighborhoods will be only marginally higher than during the background days (e.g., the storms of February 22, April 7, and May 5). In contradiction of this trend, however, PM_10_ concentrations during the storm of March 2 were elevated to the excessively high level of 800 μg m^-3^, an outcome possibly due to the storm’s unusual strength: the strongest storm of the season, it was able to transport particles through the city much more efficiently, resulting in the inundation of neighborhoods closer to the leeward side of Beer-Sheva with large concentrations of particles.

The differences found in PM_2.5_/PM_10_ ratios between storms are indicative of the dominant PM fraction for each specific storm. Thus, the ratios of ~0.75 and 0.88 from the storms of April 7 and February 22, respectively, indicate dust storms dominated by fine particles, while storms dominated by coarser particles (e.g., PM_2.5–10_) will have smaller ratios closer to 0.5. Although in this study, the observed differences in PM_2.5_/PM_10_ ratios between the measuring points for each specific storm are small (except for the mild storm of April 7), earlier studies of urban PM_2.5_/PM_10_ ratios have typically found considerable spatial variability [60–62]. Moreover, in their study in the Netherlands, Burton et al. [63] reported that the chemical composition of PM_10_ may differ spatially due to the contributions of local sources emitting aerosols in the coarser fraction of PM_10_. Nevertheless, our findings of a strong correlation between PM_2.5_ and PM_10_ values for the different measurement points and the almost constant PM_2.5_/PM_10_ ratio between storms indicate that city-wide spatial variability during dust episodes is negligible and that local dust sources make only minor contributions to PM ratios. These findings of that neighborhood-specific characteristics or anthropogenic activities have only minor effects on PM ratios suggest that in cities with characteristics similar to those of Beer-Sheva, the concentration of either fraction at any site can be reliably estimated given the concentration of the other fraction.

### Specific PM distributions during dust storms

Fig 7 shows that for the total dust period, in general, PM levels measured in the eastern parts of the city were above 1000 μg m^-3^, while those measured on the western side were extremely high, above 1700 μg m^-3^. This pattern derives from the typically westerly trajectories of most storms, which resulted in a buildup of particles on the western side of the city. However, measurement points located along the city edges or outside of residential areas exhibited lower (yet still high) PM concentrations, a finding assumed to be the result of the better ventilation of these areas compared to points deep within the city or in more built-up sections.

## Conclusions

Quantitative data on dust storm-derived particulate matter from arid areas and information on the spatial and temporal variations in arid cities is sparse. Existing PM distribution studies are typically large-scale, they focus on PM generated by anthropogenic activities, and they are limited to areas where monitoring stations are available. Therefore, this study used the city of Beer-Sheva, which has a semi-arid climate and is subjected to low anthropogenic effects, to study PM spatial variations generated by natural dust events.

The results of this study showed small spatial variability during the different periods of the year (i.e., dust vs. non-dust). Moreover, they also showed that the semi-arid climate had a stronger regional impact than did potential local emissions or removal processes. From the point of view of an efficient use of financial and personal resources, the number of collocated measurements at sites in a monitoring network can be kept quite limited in a low anthropogenic area. In addition it is possible to predict areas with higher values given the dust event trajectory and dust intensity. The values presented in the study are very high and such exposure may cause extreme health effects. These PM values are caused solely by natural dust outbreaks. Beer-Sheva is a special case where anthropogenic activity is low yet most of the cities are also exposed to anthropogenic sources and together with PM derived from dust storms, PM values can reach dangerously high values- 30 times higher than WHO guidelines.

To the best of our knowledge, this is the first study to assess the spatial distribution of dust-derived PM concentrations on an urban scale in an arid environment. A better understanding of the variations in pollution levels from difference sources, including natural ones, on a smaller scale can lead to better predictions about exposure risks. This is especially important in areas exposed to high levels of natural air pollution and that lack monitoring systems.
